# Ras Conformational Switching: Simulating Nucleotide-Dependent Conformational Transitions with Accelerated Molecular Dynamics

**DOI:** 10.1371/journal.pcbi.1000325

**Published:** 2009-03-20

**Authors:** Barry J. Grant, Alemayehu A. Gorfe, J. Andrew McCammon

**Affiliations:** 1 Department of Chemistry and Biochemistry and Center for Theoretical Biological Physics, University of California San Diego, La Jolla, California, United States of America; 2 Howard Hughes Medical Institute, University of California San Diego, La Jolla, California, United States of America; 3 Department of Pharmacology, University of California San Diego, La Jolla, California, United States of America; University of Houston, United States of America

## Abstract

Ras mediates signaling pathways controlling cell proliferation and development by cycling between GTP- and GDP-bound active and inactive conformational states. Understanding the complete reaction path of this conformational change and its intermediary structures is critical to understanding Ras signaling. We characterize nucleotide-dependent conformational transition using multiple-barrier-crossing accelerated molecular dynamics (aMD) simulations. These transitions, achieved for the first time for wild-type Ras, are impossible to observe with classical molecular dynamics (cMD) simulations due to the large energetic barrier between end states. Mapping the reaction path onto a conformer plot describing the distribution of the crystallographic structures enabled identification of highly populated intermediate structures. These structures have unique switch orientations (residues 25–40 and 57–75) intermediate between GTP and GDP states, or distinct loop3 (46–49), loop7 (105–110), and α5 C-terminus (159–166) conformations distal from the nucleotide-binding site. In addition, these barrier-crossing trajectories predict novel nucleotide-dependent correlated motions, including correlations of α2 (residues 66–74) with α3-loop7 (93–110), loop2 (26–37) with loop10 (145–151), and loop3 (46–49) with α5 (152–167). The interconversion between newly identified Ras conformations revealed by this study advances our mechanistic understanding of Ras function. In addition, the pattern of correlated motions provides new evidence for a dynamic linkage between the nucleotide-binding site and the membrane interacting C-terminus critical for the signaling function of Ras. Furthermore, normal mode analysis indicates that the dominant collective motion that occurs during nucleotide-dependent conformational exchange, and captured in aMD (but absent in cMD) simulations, is a low-frequency motion intrinsic to the structure.

## Author Summary

The Ras family of enzymes mediate signaling pathways controlling cell proliferation and development by cycling between active and inactive conformational states. Mutations that affect the ability to switch between states are associated with a variety of cancers. However, details of how the structural changes occur and how mutations affect the fidelity of this process remain to be determined. Here we employ an advanced computational technique, termed accelerated molecular dynamics, to characterize structural transitions and identify novel highly populated transient conformations. Several spatially distant structural regions were found to undergo correlated motions, highlighting a dynamic linkage between the sites of enzymatic reaction and the membrane-interacting C-terminus. In addition, our results indicate that the major motion occurring during the conformational exchange is a low-frequency motion intrinsic to the structure. Hence, features of the characterized transitions likely apply to a large number of structurally similar but functionally diverse nucleotide triphosphatases. These results provide fresh insights into how oncogenic mutations might modulate conformational transitions in Ras.

## Introduction

Ras proteins are guanine nucleotide-dependent conformational switches that couple cell-surface receptors to signaling pathways that mediate cell proliferation, growth and development [1]. Signal propagation through Ras is mediated by a regulated GTPase cycle that induces distinct conformations with different affinities for downstream effectors. The binding of GTP switches Ras to an “active” effector interacting form. Subsequent GTP hydrolysis returns Ras to the “inactive” GDP-bound form. Two types of regulatory proteins enhance the intrinsically low rates of these processes. GTPase activating proteins (GAPs) promote GTP hydrolysis, whilst guanine nucleotide exchange factors (GEFs) promote GDP release and regeneration of the active GTP-bound state. Mutations that lead to deregulated Ras activity are found in over 25% of human tumors [2]. These autonomously active variants are insensitive to the action of GAPs resulting in uncontrolled cell growth. The current work aims to better understand the basis of these oncogenic transformations by deciphering how Ras changes its structure as it executes its enzymatic cycle and how the fidelity of this process is affected by mutations.

Conformational changes and oncogenic mutations are largely concentrated in the vicinity of the nucleotide binding site, including the so-called switch regions SI (residues 25–40) and SII (residues 57–75). Of particular note are the conserved SI threonine (residue 35) and SII glycine (residue 60) which converge to form hydrogen bonds with the γ-phosphate of GTP (effectively ‘closing’ the nucleotide binding pocket). In the absence of the γ-phosphate (or a suitable analogue such as aluminium fluoride (AlF3)) the switch regions display fewer structural contacts to the nucleotide and reside in a more ‘open’ conformation. This observation has been likened to a loaded spring, where release of the γ-phosphate after GTP hydrolysis allows the switch regions to relax into their ‘open’ GDP-bound conformations [3]. Crystallographic and spectroscopic studies have indicated that the switch regions exhibit substantial mobility both within and between different nucleotide states [3]–[10]. However, a detailed sequence of events and the mechanism by which certain mutations affect key dynamic rearrangements remains elusive.

In the present study we employ simulation approaches to perform a detailed characterization of the dynamics of nucleotide-dependent conformational transitions. Previous unbiased molecular dynamics (MD) simulations were restricted to characterizing fluctuations within individual nucleotide states [11],[12]. As a result, external biasing forces (e.g., via targeted molecular dynamics) [13],[14] were required to characterize the conformational exchange between nucleotide states. However, sampling by these methods is biased along narrow channels whose transition states may be of unrealistically high energy (100 and 120 kcal/mol compared to estimates of ∼22 kcal/mol [15]). These findings highlight the need for new simulation approaches to probe bias-free transitions. Recently we reported the observation of spontaneous nucleotide-dependent transition during unbiased MD simulation of the oncogenically active G12V variant [16]. This study suggested the existence of a lower thermally accessible energetic barrier between inactive and active states of this variant that renders it prone to adopt an active conformational state. Here we extend this work with a multi-scale simulation approach employing classical and accelerated molecular dynamics (cMD and aMD) [17],[18] along with normal mode analysis (NMA) to study both wild-type and mutant transitions. Multiple-barrier-crossing aMD trajectories are used to characterize the reaction paths of the transitions. Simulated conformations are evaluated by comparison to the distribution of Ras's crystallographic conformers. The application of aMD allows the observation of nucleotide-dependent conformational transitions for wild-type Ras that are practically impossible to see with cMD. Furthermore, NMA indicates that the dominant collective motion that occurs during nucleotide-dependent conformational exchange, and captured in aMD simulations, is a low-frequency motion intrinsic to the structure. A number of highly populated intermediate conformations are characterized and their relation to available experimental structures discussed. Finally, the pattern of correlated motions in the current simulations reveals nucleotide-dependent differences of possible functional significance for membrane association.

## Results/Discussion

Simulations were conducted with starting structures corresponding to wild-type GDP, wild-type GTP and mutant GDP states of Ras. Each of these systems was simulated with a bound GDP and GTP. Both classical and accelerated MD (cMD and aMD) simulations were performed with explicit solvent for 60 nanoseconds. In addition to conventional structural analysis, which assessed the stability of Ras during the various simulations (see Text S1, Figure S1, and Table S1), principal component analysis (PCA) was used to relate the conformational sampling in the various simulations to available crystallographic structures. To this end we employ our previously reported PCA based mapping of available crystallographic structures [16]. This analysis clearly distinguished distinct conformational clusters representing wild-type GDP, wild-type GTP and a number of minor clusters populated by switch and P-loop mutants (Figure S2). Together, these crystallographic structures provide invaluable landmarks against which simulation results may be compared.

**Figure 1:**
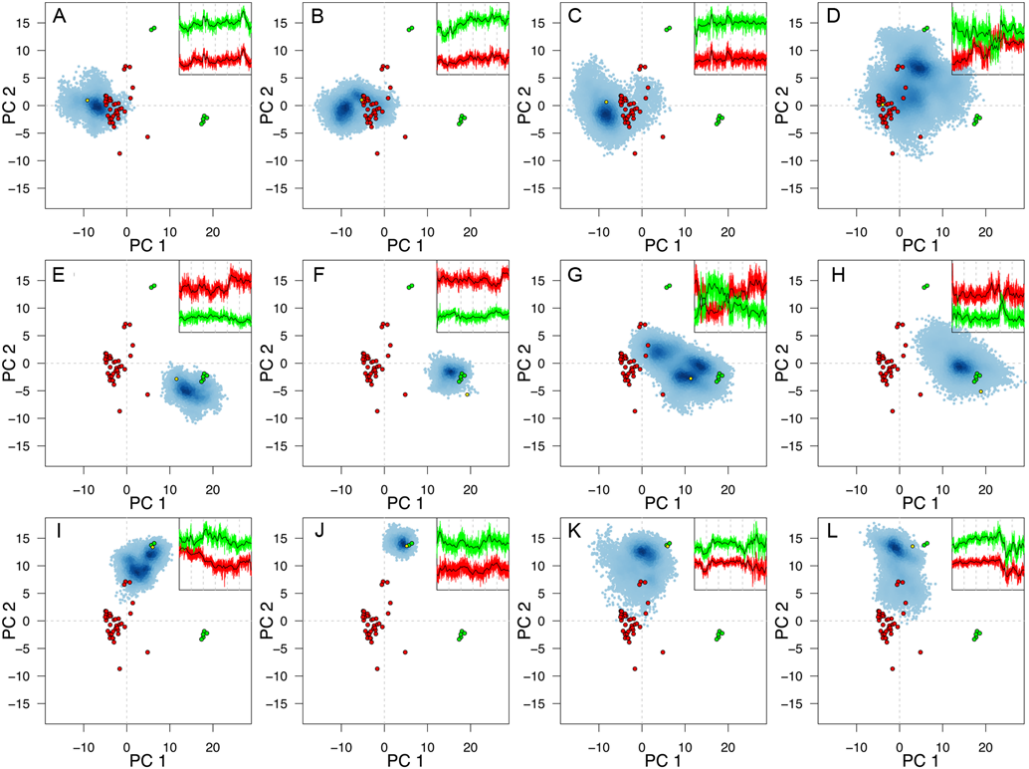
Conformational sampling in cMD and aMD simulations: projection of transient conformers onto the principal components obtained from analysis of Ras's crystallographic structures. Crystallographic GTP conformers are colored red whilst GDP conformers are colored green. The distribution of MD conformers is depicted with density-shaded blue points. Each row corresponds to a single initial conformation, namely: (A–D) wtGTP, (E–H) wtGDP and (I–L) mutantGDP. cMD simulations are depicted in the two left panels (A, B, E, F, I, J) whilst aMD simulations are depicted in the two right panels (C, D G, H, K, L). Simulations were performed with bound GTP (A, C, E, G, I, K) and GDP (B, D, F, H, J, L). Inserts show distances between instantaneous trajectory conformations and the centroids of the main GTP and GDP crystal structure clusters in red and green respectively (see Methods for further details).

Figure 1 shows the conformational space sampled by simulations projected onto the lowest PCs determined from the distribution of X-ray structures. These projections illustrate the correspondence of the crystal structure distribution and those adopted during simulations. Also shown, in terms of collective coordinates, is the time evolution of the distances between GDP and GTP conformation cluster centroids for each trajectory. All aMD simulations were found to have significantly larger fluctuations in the plane defined by the lowest PCs, indicating that the main collective displacements in the crystal structure cluster are accessible during aMD simulations (Figure 1C, 1D, 1G, 1H, 1K, and 1L and Table S2). In contrast, cMD simulations display a more limited sampling (Figure 1A, 1B, 1E, 1F, and 1J). A notable exception is the cMD simulation of the oncogenic G12V variant (Figure 1I), where the introduction of GTP into an initially GDP-bound structure induced evolution towards the cluster populated by the experimental GTP-like conformers. Additional cMD simulations of this varient with different starting conditions consistently sampled two distinct regions: one near the cluster containing the inactive form of the G12V variant, and another close to the cluster of the active structures, suggesting a spontaneous inactive to active transition.

Interestingly, aMD simulations carried out to further probe the apparent low activation barrier of the G12V variant (Figure 1K and 1L) display a similar range of sampling for both GDP and GTP-bound systems. Note that cMD simulations of this system with a bound GDP (Figure 1J) display a similar pattern of sampling to all other cMD simulations (with simulated conformers being largely restricted to the vicinity of the starting structure (Figure 1A, 1B, 1E, and 1F)). Implementing a range of boost-valued aMD simulations (see Figure S3) indicated that employing half the boost required for transition of wild-type systems (discussed bellow) was sufficient to achieve similar G12V transitions. This is consistent with the existence of a comparatively low activation barrier, even in the presence of GDP, and again highlights the intrinsic susceptibility for activation of this oncogenic mutant [16]. Furthermore, the overall agreement between the current and the previous cMD simulations, carried out with the AMBER and CHARMM force fields respectively, demonstrates that these results are robust and independent of simulation details.

In contrast to the absence of evident transitions during cMD for wild-type Ras, notable transitions are sampled during aMD for systems with a swapped nucleotide (i.e. GTP inserted into a starting GDP structure and *vice versa* (Figure 1D, 1G, and 1K)). Furthermore, the densities of populations for these simulations indicate that several highly populated intermediate conformations were sampled. In summary, the current analysis establishes the relevance of aMD simulations for investigating nucleotide-dependent conformational transitions. Subsequent discussion will therefore concentrate on further analysis of these aMD simulations of wild-type Ras that sampled the GDP-to-GTP (forward) and GTP-to-GDP (backward) transitions.

### Nucleotide-Dependent Conformational Transitions

**Figure 2:**
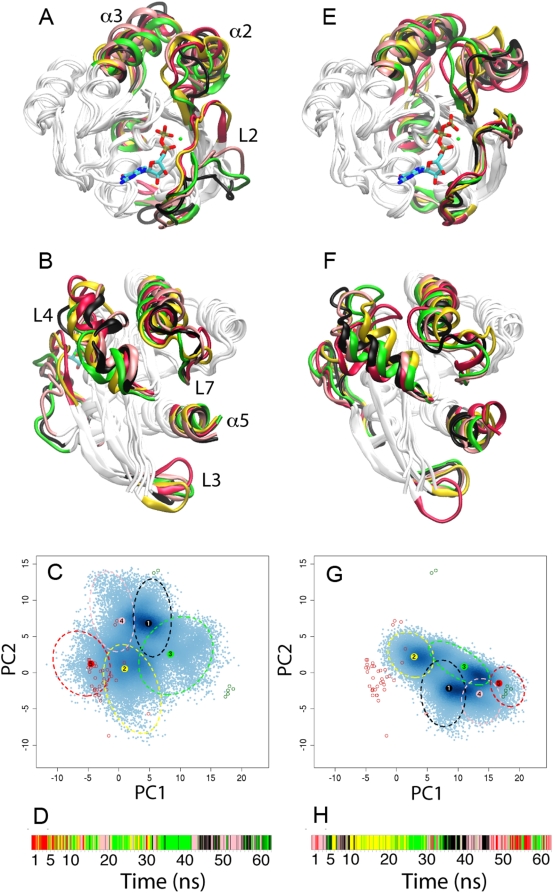
Clustering of wild-type GTP with bound GDP (A–D) and wild-type GDP with bound GTP (E–H) aMD trajectories. Front and back views of representative structures obtained from hierarchical clustering (A, B, E and F). In each case the most populated cluster representative is shown in black (representative of 30.26% and 28.56% of their respective trajectory conformers in each simulation), with subsequent clusters in yellow (23.22% and 27.91%), green (21.3% and 21.02%), pink (19.29% and 16.34%) and red (5.94% and 6.17%). PC projection plots with cluster ellipsoid hulls i.e. the ellipsoid of minimum volume such that points from a given cluster lie inside ellipsoid boundaries (C and G). Trajectory timeline colored according conformational cluster (D and H).

Clustering of trajectory conformers was used to visualize the dominant conformations sampled by each simulation (Figure 2 and Table 1). The most populated cluster in the wild-type GDP-to-GTP transition (black in Figure 2E–H and Figure S4) corresponds to the lower of the three basins in Figure 1G and overlaps with the dominant conformation sampled during cMD simulations of the same system (Figure 1E). Its overall structure is intermediate between GDP and GTP states (RMSD of 1.3 Å from both GDP and GTP representatives, PDB codes 4q21 and 1qra respectively). Members of this cluster have an intermediate α2 orientation and a closed active site loop2-SI and loop4-SII that more closely resembles the GTP configuration. The second most populated cluster (yellow in Figure 2E–H) has more distinctive GTP like characteristics (RMSD of 1 Å from GTP and 1.6 Å from GDP representatives) including a closed loop2-SI and loop4-SII active site and a reoriented GTP-like α2 helix. Interestingly, the PC projection (Figure 2G) and RMSD values (minimum value 0.4 Å) indicate that members of cluster 2 closely resemble the crystallographic GTP-bound A59G structure (PDB code 1lf0). This structure has been suggested previously to be an intermediate and is characterized by a GTP-like SII conformation and a SI conformation that has undergone partial transition with the side-chain of Y32 adopting an orientation that is intermediate between that in wild-type GDP and GTP crystallographic structures [6],[7],[16] (see Figure S2). The third cluster (green in Figure 2E–H) is again equidistant from GDP and GTP states (RMSD of 1.3 to 1.4 Å) and, similar to cluster 1, is characterized by an intermediate α2 orientation. However, unlike cluster 1, loop2-SI and loop4-SII resemble the open GDP conformation. Clusters 4 and 5 are closer to the GDP conformation (with RMSDs of 1 and 0.3 Å) than to the GTP conformation (with RMSDs of 1.4 and 1.7 Å). However, cluster 5 has distinct conformations of loop3 and the C-terminal portion of α5.

**Table 1:** Trajectory cluster minimum RMSD from GDP, GTP A59G and Y32C crystal structure representatives.

		RMSD from Representative Crystal Structure†			
System	Cluster No.	GTP	GDP	A59G	Y32C
**wtGDP-GTP**	**1**	1.32	1.31	1.32	1.38
	**2**	1.02	1.57	0.37	1.38
	**3**	1.41	1.33	1.41	1.45
	**4**	1.38	1.01	1.37	1.52
	**5**	1.73	0.32	1.61	1.78
**wtGTP-GDP**	**1**	1.62	1.91	1.64	1.73
	**2**	0.94	1.49	1.06	1.26
	**3**	1.43	1.07	1.50	1.64
	**4**	1.30	1.84	1.31	1.39
	**5**	0.82	1.58	1.06	1.19
**Reference**	**GTP**	0	1.64	0.94	1.04
	**GDP**	1.64	0	1.62	1.81
	**A59G**	0.94	1.62	0	0.68
	**Y32C**	1.04	1.81	0.68	0

**†:** GTP, GDP A59G and Y32C representatives correspond to PDB entries 1qra, 4q21, 1lf0, and 2cl7.

The GTP-to-GDP (backward) transition sampled a number of distinct conformational states (Figure 1D and Figure 2A–D). Clusters 5 and 2 have a similar loop2-SI conformation and resemble the starting GTP state (RMSD values of 0.9 and 0.8 Å, respectively). A major difference between clusters 2 and 5 is the orientation of loop3. Furthermore, conformations in cluster 2 project to a similar region of PC space to those in the second cluster of the forward GDP-to-GTP transition described above. The remaining clusters (black, green and pink in Figure 2A–D) have a strikingly open loop2-SI conformation, similar to that observed in crystal structures 1×cm, 1bkd, 1nvv, chain R of 1nvu, chain R of 1nvx, and chain B of 1×d2. Cluster 3 most closely resembles the GDP state both in terms of its PC projection (Figure 2C) and RMS distance (1.1 Å). Clusters 1 and 4 project close to the GTP-bound crystallographic structures in which Y32 has been replaced by a Cys-chromophore. However, RMSD measurements of over 1.4 Å indicate features distinct from Y32 mutants and wild-type GDP/GTP states. Taken together, these results suggest that in addition to several distinct conformations that were not observed previously, both the forward and backward transitions pass through a common intermediate similar to that captured in the A59G crystal structure.

The temporal evolution of cluster membership in each trajectory (Figure 2D and 2H) indicates that the first 4 to 5 ns remain relatively close to the starting structure (with conformations classified as cluster 5 or 4). This is followed by multiple transitions between states with periods of sampling where one conformation predominates. For example, conformations from the 10 to 25 ns period of the GDP-to-GTP trajectory reside predominantly in the GTP-like cluster 2. Similarly, conformations from the 33 to 42 ns period of the GTP-to-GDP trajectory are classified as the more GDP-like cluster 3. These single cluster blocks are interspersed with periods of rapid interconversion between clusters, for example the 30 to 35 ns portion of the GDP-to-GTP trajectory (Figure 2H). Interestingly, the GDP-to-GTP trajectory evolves toward a GTP-like cluster 2 conformation and then transitions back to a intermediate cluster 3 conformation before returning back via clusters 4 and 5 (see Videos S1 and S2). This behavior is consistent with the presence of a higher barrier between cluster 2 and the main crystallographic GTP cluster, which we fail to cross, than exists between the intermediate conformations contained within our clusters. The high level of inteconversion between these clusters also suggests that they are energetically relatively close to one another.

Analysis of the calculated structures indicates that certain side chain reorientations, diagnostic of GTP and GDP crystallographic states [6],[7],[13],[16], have only been partially realized in our simulations. We speculate that while the topology of the backbone provides the conformational blue print, specific side-chain interactions are required to stabilize the canonical GTP and GDP states. The apparent barrier between cluster 2 and the main crystallographic GTP cluster would therefore result from the energetic cost of reorganizing these side chains. A typical example is Y32, whose orientation in the simulated intermediates is neither fully solvent exposed nor hydrogen bonded with Y40 as in the main GTP and GDP crystallographic clusters respectively [16]. Rather we note its similarity to that found in the crystal structures 1gnr and 1gnq.

### Correlated Motions and the Effect of Nucleotide Exchange on Dynamics

**Figure 3:**
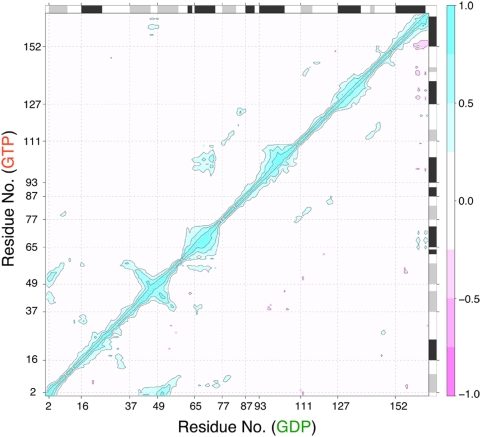
Residue-residue plot of correlated motions. The extent of correlation for all residue pairs (of Cα atomic displacement) during selected portions of the wild-type GTP (upper triangle) and wild-type GDP (lower triangle) Ras aMD simulations. The color scale runs from pink (for values ranging between −1 to −0.75), through white (−0.25 to 0.25) to cyan (0.75 to 1). Negative values are indicative of displacements along opposite directions, namely anticorrelated motions, whereas positive values depict correlated motions occurring along the same direction. Major secondary structure elements are indicated schematically with helices in black and strands in gray.

To examine whether the motions of one residue are related to the motions of another (distant) residue, the correlation of the displacements of all residue pairs were determined (Figure 3). As expected, the strongest positive correlations exist between covalently bonded residues and those residing within secondary structure elements (see Figures S5 and S6 for reference contact maps). Moving up the diagonal, the first area of notable correlations corresponds to helix α1 (residues 16 to 25) with loop2 (residues 26 to 40). The next area of significant correlation corresponds to the β2-loop3-β3 region (residues 38 to 57). The consistent appearance of correlated motions for these regions in each simulation and the cross-correlation (off-diagonal peak) with β1 (residues 2 to 10) highlights the subdomain-like structure and dynamics of these three N-terminal strands. The remaining strands, β4 to β6, display consistent positive cross-correlations with each other but not with strands β1 to β3. Note the off-diagonal peaks for β4 (residues 77 to 83) with β5 (residues 111 to 115) and β5 with β6 (residues 141 to 144). Moving further up the diagonal the next major correlations correspond to the SII region, encompassing loop4 and helix α2 (residues 58 to 74).

Perhaps the most interesting feature of the plot is the pattern of correlation between α2 and α3-loop7 (residues 66 to 74 and 93 to 110). This feature is most evident in GTP-bound simulations and is largely absent in GDP-bound simulations. This pattern is particularly noteworthy as GDP-to-GTP aMD simulations exhibit these correlations only in portions of the trajectory that reside in a GTP like conformation (i.e. after the transition from GDP to GTP, see Figure 2G and 2H). It appears that the large rearrangement of helix α2 during the transition brings it into closer register with α3, hence facilitating the correlated motions of these regions. Furthermore, the correlations of these regions in the GTP-to-GDP aMD simulation reduce gradually as the conformation of α2 evolves toward a more GDP like state. These data thus show a novel GTP-dependent correlated motion in Ras that has functional implications (see below). Additional off-diagonal peaks include loop2 with loop10 (residues 26 to 37 and 145 to 151) and loop3 with α5 (residues 46 to 49 and 152 to 167). As discussed below loop3 and α5 are connected via several salt bridges whilst both loop2 and 10 directly interact with the bound nucleotide. These newly identified coupled motions suggest a dynamic linkage between the N-terminal nucleotide-binding subdomain and the C-terminal subdomain whose downstream residues are responsible for membrane binding.

The dissection of the catalytic domain into two lobes or subdomains based on the correlated motions of the central β-strands is consistent with the localized nature of sequence variation between Ras isoforms. As previously noted [16], lobe 1 (residues 1–86) is strictly conserved in sequence and encompasses the P-loop and the switch regions; whilst lobe 2 (residues 87–171) contains amino acid variations that define functionally distinct H, N and K-ras isoforms [16]. As isoform-specific properties include differences in nucleotide-state dependent membrane localization [19]–[21], the segregation of both sequence variation and correlated motions implies that communication between lobes is likely to be of functional significance. The covalent connection between lobes is made by helix α2 of the SII region, which is the major dynamic element of the Ras structure. The current cross-correlation analysis indicates the existence of three additional non-covalent communication routes between lobes including loop3 to α5, loop2 to loop10 and α2 to α3-loop7. We speculate that residues at each of these sites may be important for nucleotide-dependent modulation of membrane attachment and lateral segregation by linking the switching apparatus to the membrane interaction apparatus. Indeed, alanine substitution of loop3 residues D47 and E49 produced a variant that is hyperactive in MAPK-signaling [22]. These loop3 residues, together with their α5 salt bridge partners (R161/R164), have been shown to modulate the nucleotide-dependent membrane association of H-ras [22],[23]. The other regions highlighted in the current study have thus far received little attention but likely warrant further investigation.

### Normal Mode Analysis

**Figure 4:**
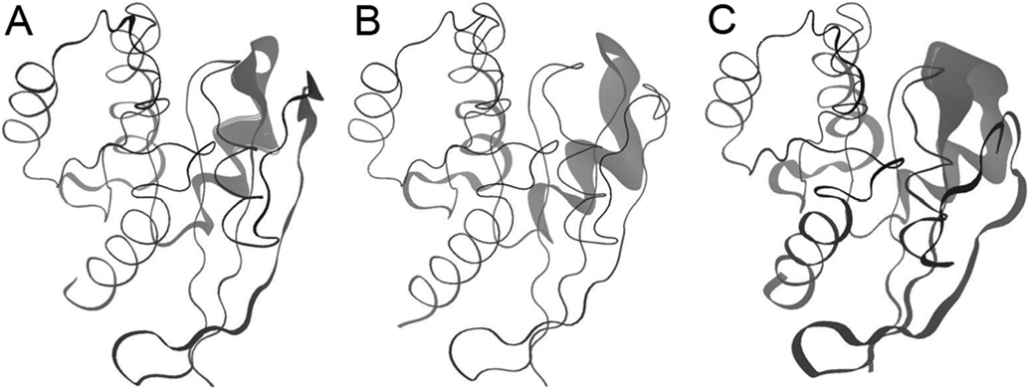
Visualization of dominant motions obtained from (A) PCA of Ras crystal structures, (B) NMA of wild-type GDP Ras, and (C) aMD of wild-type GDP Ras.

In an effort to further understand the physical basis of the observed motions upon nucleotide exchange, we analyzed available structures with a simplified elastic-network normal mode method [24]. The elastic network approach has the advantage that a single model, expressed in terms of Cα coordinates, leads to an objective expression of possible protein dynamics in terms of a superposition of collective normal mode coordinates [25]. Consistent with previous studies on a range of systems [26], we note that only the “open” wild type GDP conformation yielded large overlap values between the crystal structure PCs and the low-frequency normal modes (overlap between NMA mode 1 and X-ray PC 1 of 0.57). Furthermore, the structural mobility predicted by NMA is very similar to that obtained from aMD simulations (overlap between GDP-to-GTP trajectory PC 1 and X-ray PC 1 of 0.69; Figure 4 and Table S2). This result implies that low-frequency global motions that are intrinsic to the open structure likely facilitate the observed conformational transitions to the closed GTP state. In other words, the fact that low frequency normal modes qualitatively capture the differences between available crystal structure conformations and have high overlap with the eigenvectors obtained from aMD simulations suggests that nucleotide-dependent dynamics is facilitated by the low frequency, global motions that are intrinsic to the structure. The aMD results discussed above indicate that the nature of the bound nucleotide attenuates these intrinsic motions.

### Conclusions

We have characterized the spontaneous transition between nucleotide-dependent conformational states of wild-type Ras with cMD, aMD and NMA. These functionally important transitions, achieved for the first time for wild-type Ras, are practically impossible to observe with cMD. Furthermore, NMA indicates that the dominant collective motion that occurs during these transitions is a low-frequency motion intrinsic to the structure.

Mapping the reaction path sampled by aMD onto a PCA basis set derived from the distribution of crystallographic structures enabled identification of intermediate structures with unique switch orientations and/or distinct loop3, loop7 and α5 C-terminus conformations. Intriguingly, several of the highly populated intermediates have a close correspondence to known G59A and Y32C crystallographic conformers, both of which have been suggested to be intermediate structures [6],[16]. The emergence of these conformations along with additional novel intermediates highlights the utility of aMD simulations to reliability sample conformational transitions. Furthermore, the current results imply that the G59A and Y32C variant conformations are accessible to wild-type Ras and that these mutations result in perturbations that localize the average structure at these intermediate positions. The functional relevance of these intermediates is reinforced by kinetic studies of G59A that indicated a reduced rate of nucleotide exchange; this has been linked to the need for tight nucleotide coordination during structural changes [6],[16]. It will be interesting to see if our newly identified intermediates, some of which differ between forward and backward transitions, also have distinct kinetic behaviors related to nucleotide exchange and phosphate release.

The pattern of correlated motions revealed by these simulations predicts novel nucleotide-dependent motions of potential significance in the signaling function of Ras. These include correlations of α2 with α3-loop7, loop2 with loop10 and loop3 with α5. Such dynamic linkages between the switching apparatus and the membrane interacting C-terminal region leads us to speculate that residues at each of these sites may be important for nucleotide-dependent modulation of membrane attachment. This is supported by recent experimental evidence for the role of loop3 residues D47 and E49 and α5 residues R161 and R164 in modulating the nucleotide-dependent membrane association of Ras [22],[23].

Finally, low frequency normal modes qualitatively capture the differences between available crystal structure conformations and have high overlap with the eigenvectors obtained from aMD simulations. This result combined with aMD observations suggests that nucleotide-dependent dynamics is facilitated by low frequency, global motions that are intrinsic to the structure and that the nature of the bound nucleotide serves to attenuate these intrinsic low-frequency motions. Furthermore, the significant similarities of aMD, NMA and crystal structure PCA motions highlight the robustness of the observed motions.

We believe that the current advanced simulation and analysis approach is equally applicable to a large number of structurally similar but functionally diverse P-loop NTPases such as kinesin and myosin. Such studies should uncover detailed dynamic behavior and help inform us about general principles and mechanisms underlying nucleotide-dependent conformational changes.

## Methods

All simulations were performed with the AMBER8 package [27] and corresponding all-atom potential function ff99SB [28]. Additional parameters for guanine nucleotides were taken from Meager at al. [29]. All analysis was carried out using the Bio3D package [30],[31].

Atomic models were prepared from three high-resolution crystal structures (PDB codes: 4Q21, 1QRA and 2Q21). These structures are representative of three distinct conformations highlighted by PCA [16]; namely: wild-type GDP, wild-type GTP and mutant GDP. Each model was simulated with bound Mg^2+^·GDP and Mg^2+^·GTP. The latter was obtained for GDP structures by the addition and local optimization of the γ-phosphate onto the GDP of the corresponding crystal structure.

### Molecular Dynamics

All MD simulations were performed using periodic boundary conditions, TIP3P water and charge-neutralizing counter ions, with full particle-mesh Ewald electrostatics. Operational parameters included a 2fs time step and a 10Å cutoff for the truncation of VDW non-bonded interactions. Constant volume heating (to 300 K) was performed over 10ps, followed by constant temperature (300 K), constant pressure (1atm) equilibration for an additional 200ps. Finally, constant pressure constant temperature production dynamics was performed with both classical and accelerated MD implementations. The SHAKE algorithm was used to constrain all covalent bonds involving hydrogen atoms.

### Accelerated Molecular Dynamics

Accelerated MD (aMD) extends the accessible time scale of conventional MD simulations by altering the underlying potential energy surface of the system under study. Acceleration stems from the addition of a non-negative boost potential that raises the energy within basins [18]. Hence a trajectory propagated on this modified surface makes transitions from state to state with an accelerated rate. Furthermore, canonical ensemble averages of the system can be obtained by reweighing each point on the modified potential by the strength of the Boltzmann factor of the bias energy at that particular point [18]. In the current study we apply the dual boost approach and corresponding potential developed previously [17],[18]. Starting structures and standard operational parameters were identical to those used for cMD. The energy level, *E*, below which the boost is applied and tuning parameter, α, that modulates the depth and local roughness of basins in the modified potential were based on an earlier work [17].

### Normal Mode Analysis

We employed the coarse-grained AD-ENM normal mode analysis approach developed by Zheng et al. [24]. AD-ENM implements a single-parameter Hookean potential, which has previously been shown to yield low-frequency normal modes that are in good agreement with those obtained from more detailed, empirical, force fields. For further details see [24],[25]

### Principal Component Analysis

PCA was employed to aid the interpretation of interconformer relationships. We utilized the previously reported PC basis set obtained from analysis of available Ras crystal structures [16]. This basis set gives a clear separation of nucleotide-dependent conformational states. Projecting the Ras crystal structures and snapshots from MD trajectories into the sub-space defined by the largest PCs (along which the crystal structure variance is largest) results in a lower dimensional representation of the structural dataset (see Figure 2 for details). The resulting low-dimensional ‘conformer plots’, succinctly reveal the nature of conformational sampling during simulations [30]. PCA was carried out on the individual trajectories using the same Cα atoms that were used in the analysis of the crystal structures. Conformer superposition was also based on the “core positions” obtained from crystal structure analysis [16].

Distances between instantaneous trajectory conformations and the centroids of the main GTP and GDP clusters, reported as inserts in Figure 1, were calculated as the Euclidean distance between projected points in five dimensional PC space. Note that five PCs account for over 81% of the variance in the original distribution and produce a more succinct distance measure than the examination of average all-atom distances. This metric aids interpretation of an otherwise noisy signal as it is derived primarily from the concerted displacement of the switch regions comprising the secondary structure elements loop2 and loop4-α2 (residues 31 to 37 and 59 to 72).

### Conformer Clustering

Structures from aMD simulations underwent average-linkage hierarchical clustering according to their pairwise RMSD distance matrix. Inspection of the resulting dendogram was used to partition structures into five dominant groups (ranked according to their populations). The closest structure to the average structure from each cluster, in terms of RMSD, was chosen as a representative for projection onto the PCA basis set described above.

### Cross-Correlation Analysis

To identify protein segments with correlated atomic motions the cross-correlation coefficient, *C_ij_*, for the displacement of all Cα atom pairs, *i* and *j*, was calculated
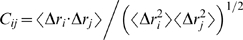
where Δ*r_i_* is the displacement from the mean position of the *i* ^th^ atom determined from all configurations in the trajectory segment being analyzed (see [32] and [33] for further details).

## Supporting Information

Figure S1 Time evolution of Cα atom RMSD from the initial structure of each simulation. Each row corresponds to a single system namely: (A and B) wtGTP, (C and D) wtGDP, (E and F) mutantGDP. Regular MD simulations are depicted in the left panel (A, C, and E,) whilst aMD simulations are depicted on the right (B, D, and F). Simulations with a bound GDP are plotted in green whilst GTP-bound systems are plotted in red. The light green and red lines correspond to the core residues used for superposition.
(2.11 MB TIF)

Figure S2 Principal component based mapping of Ras crystallographic structures. Structures are colored by nucleotide state, triphosphate in red and diphosphate in green and labeled with their PDB code where space permits. Dashed ovals represent the grouping obtained from hierarchical clustering of the projected structures in the PC1 to PC3 planes. Insert: eigenvalue spectrum detailing results obtained from diagonalization of the atomic displacement correlation matrix of Cα atom coordinates. The magnitude of each eigenvalue is expressed as the percentage of the total variance (mean-square fluctuation) captured by the corresponding eigenvector. Labels beside each point indicate the cumulative sum of the total variance accounted for in all preceding eigenvectors (1).
(0.19 MB TIF)

Figure S3 High boost value simulation of mutant GDP system with a bound GDP, see Figure 1. and main text for further details.
(2.27 MB TIF)

Figure S4 Heatmap illustrating RMSD clustering of wild-type GDP with bound GTP aMD simulation. See Figure 2E–H and methods section for further details.
(1.83 MB TIF)

Figure S5 Contact map of initial wtGTP and wtGDP Ras conformations. Residues are considered in contact when any non-hydrogen atom from a given pair of residues is separated by less than 4Å.
(1.83 MB TIF)

Figure S6 Trajectory averaged contact maps for wtGTP-GTP and wtGDP-GDP simulations. The color scale indicates the fraction of frames in which a given residue-residue contact is present.
(0.05 MB PNG)

Table S1 Selected time-averaged properties for cMD and aMD simulations†. † Values listed include average Cα atom RMSF along with Cα atom RMSD values for all and core residue subsets during each simulation. System codes are based on the starting structures of the simulations: wtGDP = GDP-bound x-ray structure from the pdb (2), code 4q21; wtGTP = GTP-bound xray structure 1qra; mutGDP = GDP bound G12V structure in 1q21. Note that the time evolution of backbone hydrogen bonds and secondary structure content also remained constant throughout all simulations (not shown).
(0.04 MB DOC)

Table S2 Comparison of crystal structure and trajectory derived eigenvectors. Inner products between the first five eigenvectors obtained from crystal structure PCA and the first ten eigenvectors obtained from PCA of individual aMD and cMD trajectories.
(0.04 MB PDF)

Text S1 Supplementary Information Text
(0.10 MB PDF)

Video S1 Conformational sampling during accelerated molecular dynamics simulation of Ras GDP with a bound GTP.
(2.56 MB MOV)

Video S2 aMD trajectory snapshots from the 5 to 30 ns portion of GDP with bound GTP trajectory. For reference, the orientation of helix alpha2 in representative GTP (red) and GDP (green) crystal structures are displayed as solid cylinders. See Video S1 and the main text for details.
(27.46 MB MOV)
