# Supplementary figures and images for: Ras Conformational Switching: Simulating Nucleotide-Dependent Conformational Transitions with Accelerated Molecular Dynamics
Source: PLoS Comput Biol. 2009 Mar 20;5(3):e1000325. doi: 10.1371/journal.pcbi.1000325 (PMC2651530; doi:10.1371/journal.pcbi.1000325)

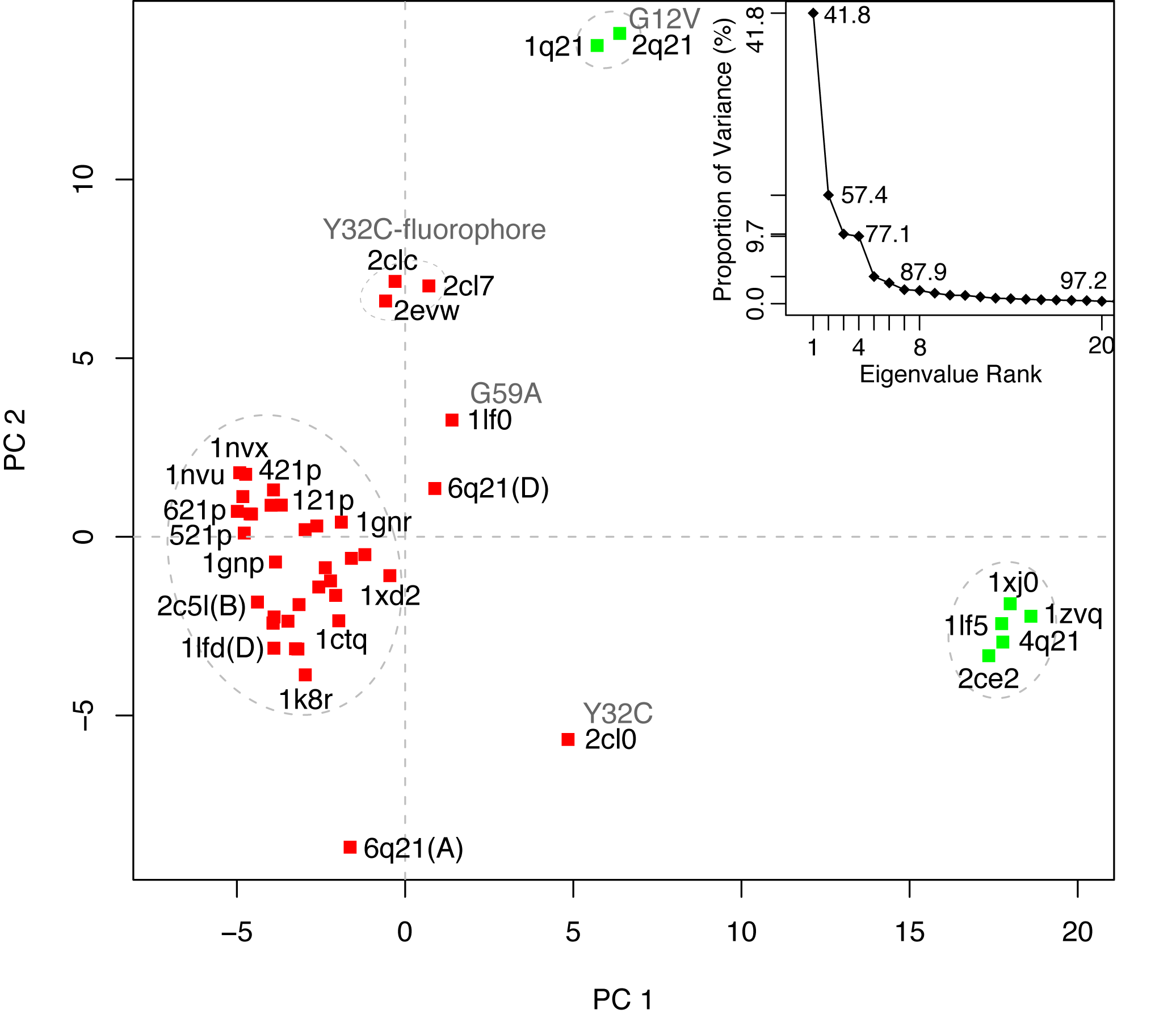

Supplement: Figure S1 — Time evolution of Cα atom RMSD from the initial structure of each simulation. Each row corresponds to a single system namely: (A and B) wtGTP, (C and D) wtGDP, (E and F) mutantGDP. Regular MD simulations are depicted in the left panel (A, C, and E,) whilst aMD simulations are depicted on the right (B, D, and F). Simulations with a bound GDP are plotted in green whilst GTP-bound systems are plotted in red. The light green and red lines correspond to the core residues used for superposition. (2.11 MB TIF) [file pcbi.1000325.s001.tif]

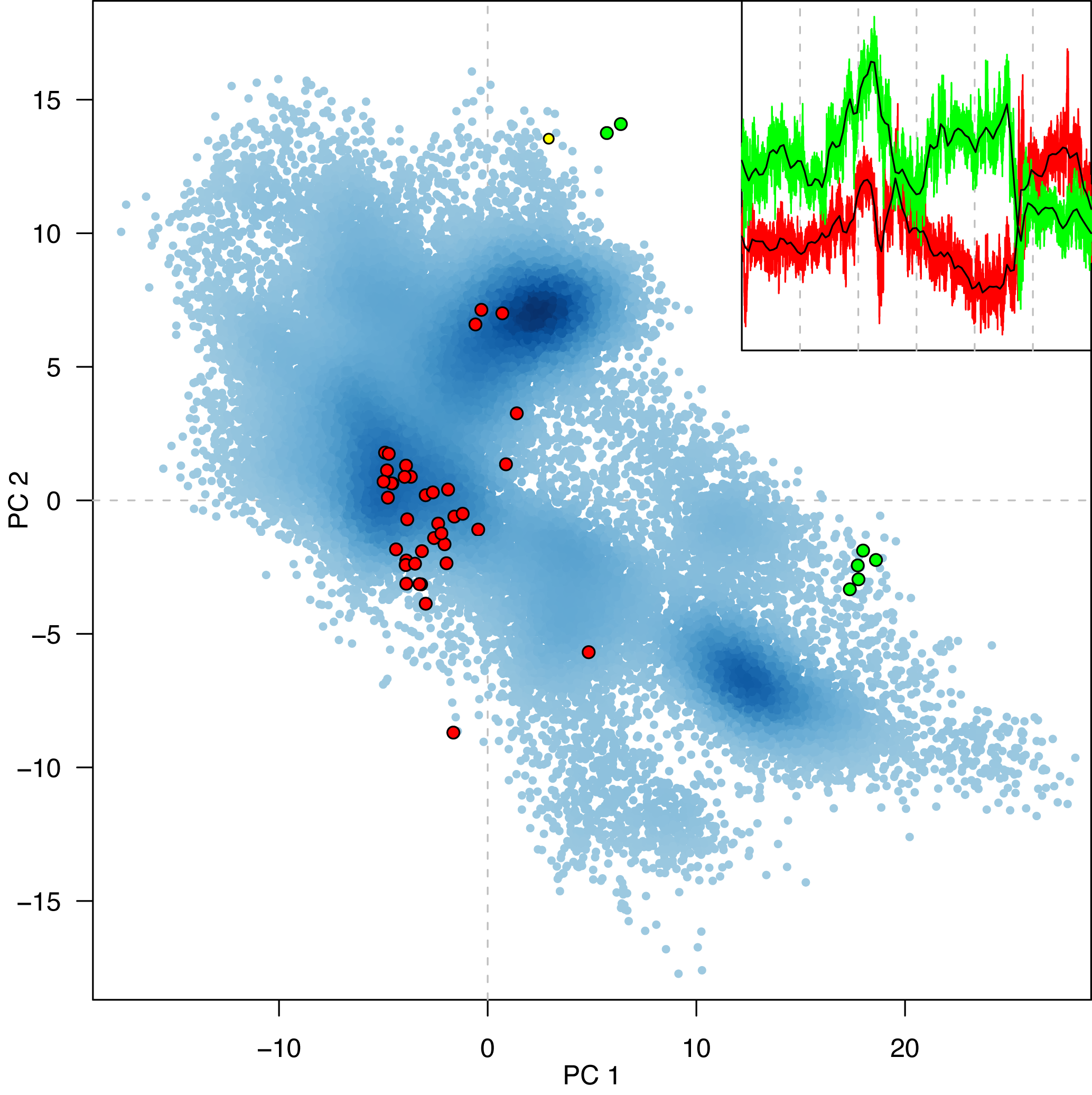

Supplement: Figure S3 — High boost value simulation of mutant GDP system with a bound GDP, see Figure 1. and main text for further details. (2.27 MB TIF) [file pcbi.1000325.s003.tif]

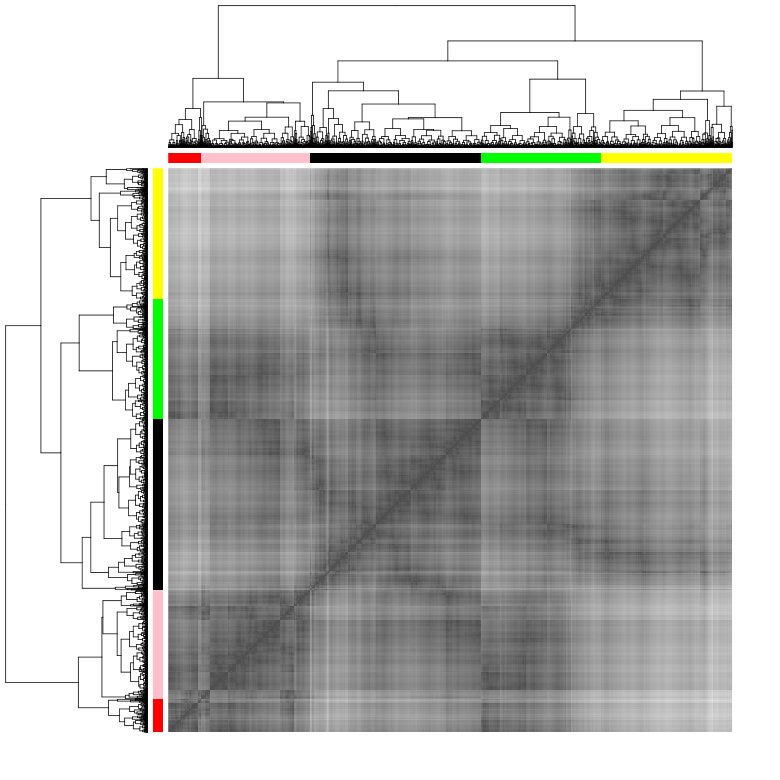

Supplement: Figure S4 — Heatmap illustrating RMSD clustering of wild-type GDP with bound GTP aMD simulation. See Figure 2E–H and methods section for further details. (1.83 MB TIF) [file pcbi.1000325.s004.tif]

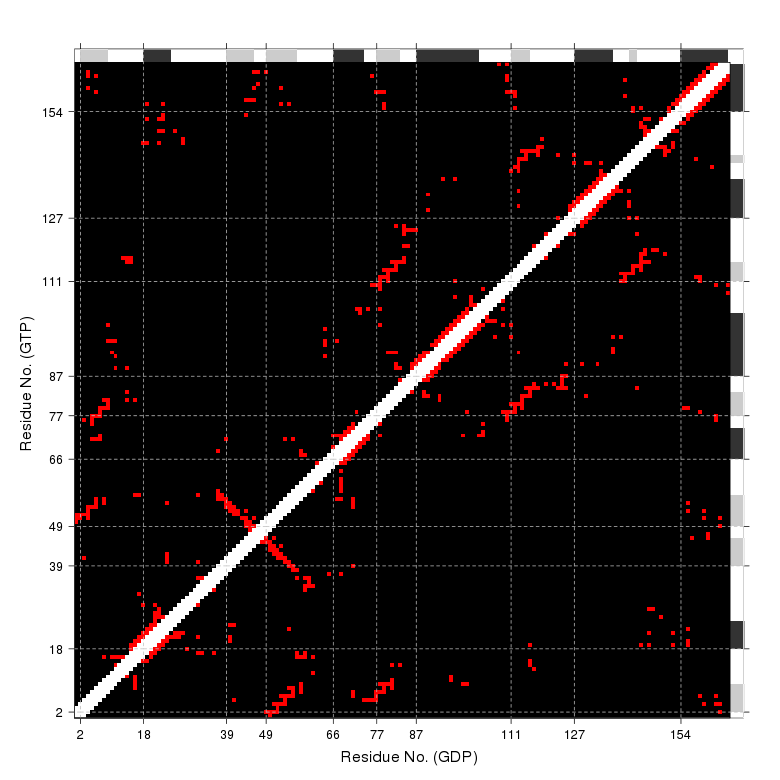

Supplement: Figure S5 — Contact map of initial wtGTP and wtGDP Ras conformations. Residues are considered in contact when any non-hydrogen atom from a given pair of residues is separated by less than 4Å. (1.83 MB TIF) [file pcbi.1000325.s005.tif]

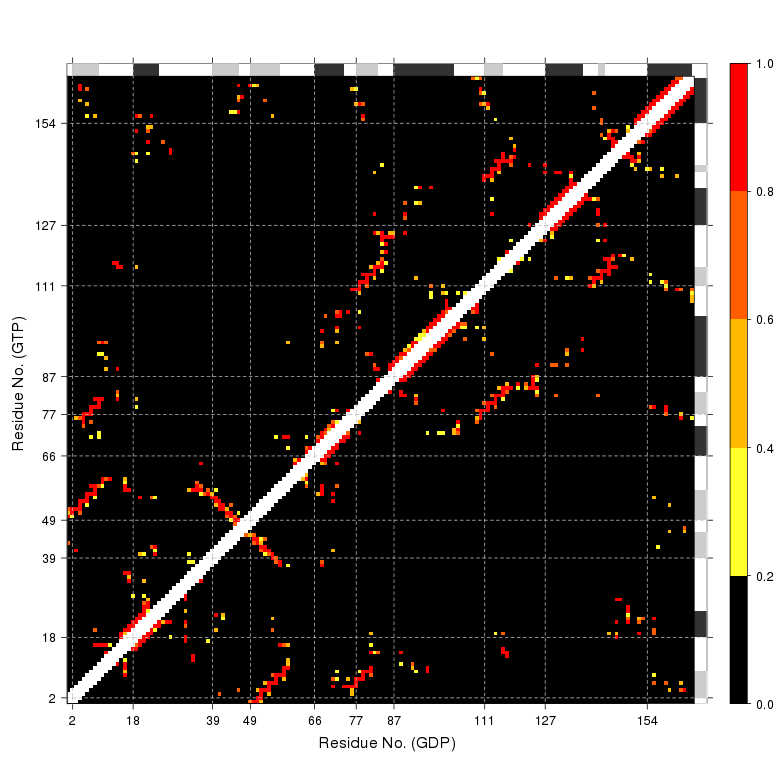

Supplement: Figure S6 — Trajectory averaged contact maps for wtGTP-GTP and wtGDP-GDP simulations. The color scale indicates the fraction of frames in which a given residue-residue contact is present. (0.05 MB PNG) [file pcbi.1000325.s006.png]
