# Supplementary material for: Ras Conformational Switching: Simulating Nucleotide-Dependent Conformational Transitions with Accelerated Molecular Dynamics
Source: PLoS Comput Biol. 2009 Mar 20;5(3):e1000325. doi: 10.1371/journal.pcbi.1000325 (PMC2651530; doi:10.1371/journal.pcbi.1000325)
Supplement: Table S1 — Selected time-averaged properties for cMD and aMD simulations†. † Values listed include average Cα atom RMSF along with Cα atom RMSD values for all and core residue subsets during each simulation. System codes are based on the starting structures of the simulations: wtGDP = GDP-bound x-ray structure from the pdb (2), code 4q21; wtGTP = GTP-bound xray structure 1qra; mutGDP = GDP bound G12V structure in 1q21. Note that the time evolution of backbone hydrogen bonds and secondary structure content also remained constant throughout all simulations (not shown). (0.04 MB DOC) [file pcbi.1000325.s007.doc]

| System | Nucleotide | RMSD | | | | **RMSF** | |
| --- | --- | --- | --- | --- | --- | --- | --- |
|  |  | **All atoms** | | **Core atoms** | | Cα-atoms | |
|  |  | **cMD** | **aMD** | **cMD** | **aMD** | **cMD** | aMD |
| wtGTP | **GTP** | **1.2 (0.2)** | **1.8 (0.3)** | **0.7 (0.1)** | **1.0 (0.2)** | **0.7 (0.4)** | 1.1 (0.8) |
|  | **GDP** | **1.5 (0.2)** | **2.5 (0.5)** | **0.8 (0.1)** | **1.2 (0.1)** | **0.8 (0.5)** | 1.4 (1.2) |
| wtGDP | **GTP** | **1.4 (0.2)** | **2.0 (0.3)** | **0.7 (0.1)** | **0.9 (0.1)** | **0.8 (0.5)** | 1.1 (0.9) |
|  | **GDP** | **1.5 (0.2)** | **2.0 (0.3)** | **0.7 (0.1)** | **0.9 (0.1)** | **0.7 (0.4)** | 1.0 (0.6) |
| mutGDP | **GTP** | **1.8 (0.2)** | **2.7 (0.4)** | **0.9 (0.1)** | **1.0 (0.1)** | **0.8 (0.6)** | 1.0 (0.8) |
|  | GDP | 1.5 (0.1) | 1.8 (0.2) | 0.8 (0.1) | 1.0 (0.1) | 0.7 (0.4) | 0.9 (0.7) |
