# Supplementary material for: Ras Conformational Switching: Simulating Nucleotide-Dependent Conformational Transitions with Accelerated Molecular Dynamics
Source: PLoS Comput Biol. 2009 Mar 20;5(3):e1000325. doi: 10.1371/journal.pcbi.1000325 (PMC2651530; doi:10.1371/journal.pcbi.1000325)
Supplement: Table S2 — Comparison of crystal structure and trajectory derived eigenvectors. Inner products between the first five eigenvectors obtained from crystal structure PCA and the first ten eigenvectors obtained from PCA of individual aMD and cMD trajectories. (0.04 MB PDF) [file pcbi.1000325.s008.pdf]

	**1**	**2**	**3**	**4**	**5**	**6**	**7**	**8**	**9**	**10**
**aMD wtGDP-GTP**										
**1**	0.69	-0.21	0.13	0.45	0.15	-0.07	0.38	0.1	0.03	0.13
**2**	0.11	-0.2	-0.29	-0.05	0.53	0.01	-0.19	-0.05	-0.05	-0.08
**3**	-0.11	0.37	0.15	0.08	-0.25	-0.23	-0.13	0.04	0.73	-0.07
**4**	-0.08	-0.19	-0.43	0.19	0.28	0.57	0.03	-0.08	-0.37	-0.03
**5**	-0.06	-0.21	0.01	-0.06	0.01	0.01	0.05	0.06	0.15	-0.38
**cMD wtGDP-GTP**										
**1**	0.34	0.33	0.1	-0.2	-0.11	-0.43	-0.08	0.05	-0.02	0.04
**2**	0.03	-0.11	0.12	0.41	-0.17	0.07	-0.08	-0.3	0.19	-0.2
**3**	0.09	-0.24	0.4	-0.05	0.04	-0.01	-0.09	-0.05	0.22	-0.1
**4**	0.17	0.27	-0.17	0.32	-0.19	-0.13	0.23	-0.07	-0.19	0.21
**5**	0.15	0.04	0.15	0.14	-0.08	0.13	0.01	0.14	-0.03	-0.04
**aMD wtGTP-GDP**										
**1**	0.52	-0.24	-0.27	-0.32	-0.07	-0.01	0.31	0.02	-0.37	0.15
**2**	0.17	0.14	0.49	0.29	0.53	-0.13	-0.1	0.3	-0.11	-0.13
**3**	-0.39	-0.25	-0.19	0.08	0.23	-0.12	-0.21	0.21	0.02	-0.09
**4**	0.06	0.35	-0.1	-0.23	0.05	-0.14	0.32	0.13	0.01	-0.3
**5**	-0.11	0.17	-0.26	-0.01	0.05	-0.06	0.06	0.07	-0.05	0.07
**cMD wtGTP-GDP**										
**1**	0.34	0.17	-0.47	0.1	0.04	0.18	-0.1	0.28	-0.22	0.15

**2**	0.11	-0.22	-0.02	-0.27	-0.42	-0.12	0.13	0.04	0.08	-0.2
**3**	0.19	0.08	-0.05	0.23	-0.32	0.02	0.1	-0.21	-0.03	-0.18
**4**	0.13	-0.26	-0.29	-0.18	0.12	0.09	0.17	0.18	-0.07	0.17
**5**	0.12	-0.11	0.03	-0.15	0.03	0.37	0.12	-0.06	0.05	-0.05
