# Supplementary material for: Ras Conformational Switching: Simulating Nucleotide-Dependent Conformational Transitions with Accelerated Molecular Dynamics
Source: PLoS Comput Biol. 2009 Mar 20;5(3):e1000325. doi: 10.1371/journal.pcbi.1000325 (PMC2651530; doi:10.1371/journal.pcbi.1000325)
Supplement: Text S1 — Supplementary Information Text (0.10 MB PDF) [file pcbi.1000325.s009.pdf]

Simulating Ras Conformational Switching: Probing Nucleotide Dependent Conformational Transitions with Accelerated Molecular Dynamics.

Barry J. Grant[a*†], Alemayehu A. Gorfe[a*†] and J. Andrew McCammon[abc]

[a] Department of Chemistry and Biochemistry and Center for Theoretical Biological Physics, [b] Howard Hughes Medical Institute, [c] Department of Pharmacology, University of California at San Diego, La Jolla, CA, 92093-0365

*Equal Contribution

Corresponding authors[†]:
Barry J. Grant and Alemayehu A. Gorfe
Department of Chemistry & Biochemistry
University of California, San Diego
9500 Gilman Drive, Mail Code 0365
La Jolla, CA 92093-0365
858-822-1469 (Office)
858-534-4974 (Fax)
bgrant@mccammon.ucsd.edu and abebe@mccammon.ucsd.edu

System Stability in cMD and aMD Simulations

The time-averaged values of various geometric properties (Table S1) indicate that both cMD and aMD simulations are free of obvious artifacts. The time evolution of the RMSD from the respective initial structures (Figure S1), are consistently higher for aMD (with a mean value over all aMD simulations of 2.1 ± 0.4 Å compared with 1.5 ± 0.2 Å for cMD). This indicates that larger displacements occurred in each of the aMD simulations, reflecting the elevated energy surface upon which aMD trajectories are propagated. Note that the RMS values obtained during aMD are similar in magnitude to the difference between various crystal structures (e.g. 2.1 Å between 6q21 chain A and 2q21). Furthermore, the core residues of the catalytic domain (1) exhibited significantly lower fluctuations (mean value of 1.0 ± 0.2 and 0.8 ± 0.1 Å for aMD and cMD), indicating that the core structure of the protein remains stable. Taken together, these data provide evidence for the stability of the catalytic domain in both cMD and aMD simulations; during each simulation it was observed that the majority of the protein remains close to the initial structure, while the flexible switch regions (discussed further in the main manuscript) change their conformation.

Additional Methods

Visual inspections and molecular figures were generated using VMD (3).

Eigenvector overlap values ranging between 0 and 1 were used to asses the degree of similarity between the direction of two eigenvectors, such as those obtained from crystal structure PCA, trajectory PCA and NMA (4):

$$O_{ij} = \frac{\left|P_i \cdot M_j\right|}{\left\|P_i\right\|\left\|M_j\right\|}$$

for example where P_i is the ith PC and M_j is the jth normal mode or PC.

References

1. Gorfe, A. A., Grant, B. J., & McCammon, J. A. (2008) Mapping the nucleotide and isoform dependent structural and dynamical features of Ras proteins *Structure* **27,** 727-735.
2. Berman, H., Henrick, K., & Nakamura, H. (2003) Announcing the worldwide Protein Data Bank *Nat Struct Biol* **10,** 980.
3. Humphrey, W., Dalke, A., & Schulten, K. (1996) VMD: visual molecular dynamics *J Mol Graph* **14,** 33-38, 27-38.
4. Tama, F. & Sanejouand, Y. H. (2001) Conformational change of proteins arising from normal mode calculations *Protein Engineering* **14,** 1-6.
